# Accounting for genomic pre-selection in national BLUP evaluations in dairy cattle

**DOI:** 10.1186/1297-9686-43-30

**Published:** 2011-08-18

**Authors:** Clotilde Patry, Vincent Ducrocq

**Affiliations:** 1 INRA, UMR 1313 Génétique Animale et Biologie Intégrative, F-78350 Jouy-en-Josas, France; 2 UNCEIA, Département de Génétique, 149, rue de Bercy, F-75595 Paris Cedex, France

## Abstract

**Background:**

In future Best Linear Unbiased Prediction (BLUP) evaluations of dairy cattle, genomic selection of young sires will cause evaluation biases and loss of accuracy once the selected ones get progeny.

**Methods:**

To avoid such bias in the estimation of breeding values, we propose to include information on all genotyped bulls, including the culled ones, in BLUP evaluations. Estimated breeding values based on genomic information were converted into genomic pseudo-performances and then analyzed simultaneously with actual performances. Using simulations based on actual data from the French Holstein population, bias and accuracy of BLUP evaluations were computed for young sires undergoing progeny testing or genomic pre-selection. For bulls pre-selected based on their genomic profile, three different types of information can be included in the BLUP evaluations: (1) data from pre-selected genotyped candidate bulls with actual performances on their daughters, (2) data from bulls with both actual and genomic pseudo-performances, or (3) data from all the genotyped candidates with genomic pseudo-performances. The effects of different levels of heritability, genomic pre-selection intensity and accuracy of genomic evaluation were considered.

**Results:**

Including information from all the genotyped candidates, i.e. genomic pseudo-performances for both selected and culled candidates, removed bias from genetic evaluation and increased accuracy. This approach was effective regardless of the magnitude of the initial bias and as long as the accuracy of the genomic evaluations was sufficiently high.

**Conclusions:**

The proposed method can be easily and quickly implemented in BLUP evaluations at the national level, although some improvement is necessary to more accurately propagate genomic information from genotyped to non-genotyped animals. In addition, it is a convenient method to combine direct genomic, phenotypic and pedigree-based information in a multiple-step procedure.

## Background

In dairy cattle, selection decisions on candidates are now widely based on Genomically Enhanced Breeding Values (GEBV) instead of Estimated Breeding Values (EBV) obtained after progeny testing. Together with the increasing availability of genotypes, further methodological developments are expected to increase the reliability of GEBV and to achieve higher genetic progress.

One challenge is to combine genomic and non-genomic information for all the animals, whether they are genotyped or not. Indeed, the number of genotyped animals is still small compared to the number of non-genotyped animals with phenotypes. Having animals with both EBV and GEBV and other animals with EBV only creates some uncertainty for breeding companies and farmers on how to optimally choose among the candidates for selection. It is also desirable to use all available information, whether genomic, phenotypic or pedigree-based, to assess the additive genetic value of any animal. Currently, there are two alternative procedures to combine data, either a multi-step procedure [1,2], which is based on selection index theory, or a single-step procedure (SSP) based on a relationship matrix that blends full pedigree and genomic information to simultaneously evaluate genotyped and non-genotyped animals [3-5]. How to correctly propagate information from genotyped to non-genotyped animals without overestimating reliabilities and without biasing breeding values remains an issue [4,6].

Including genotyped and non-genotyped animals in a single genetic analysis is also necessary to properly account for biases due to selective genotyping [7] or phenotyping [4,6,8]. The latter corresponds, for example, to young sires that are pre-selected based on genomic information: only sires with higher GEBV and hence with a higher Mendelian sampling term receive phenotypes from daughters a few years after pre-selection. BLUP (Best Linear Unbiased Prediction) assumes that Mendelian sampling terms have zero expectation [9]. Thus, genomic pre-selection (GPS) leads to biased EBV and reduced accuracy in national genetic evaluations based on a polygenic model [10]. In France, genomic evaluations became official in 2009. Since then, bulls that were pre-selected according to genomic information have been used. In 2013, the first records of their daughters will be included in the national BLUP evaluation and the resulting EBV might be biased. One concern is that biased EBV and their corresponding daughter yield deviations (DYD) may impact the estimation of SNP effects in subsequent years. This issue is also relevant at the international level, since the trade of bull semen is based on EBV from Multiple Across Country Evaluations (MACE) that are computed assuming unbiased national EBV. With genomic pre-selection more and more widely implemented, accounting for such practices is becoming very important.

Ducrocq and Liu [6] proposed a method to include genomic information in national BLUP evaluations. The approach consists of de-regressing all GEBV on which pre-selection was based, using the effective contribution of the additional genomic information as the weight. Then, all the genotyped candidates receive a pseudo-record based on genomic information to be included in the mixed model equations (MME), in addition to the actual phenotypic records. The BLUP model assumption that all sources of information on which selection is based are included is then fulfilled.

The aim of this study was to implement such a method and to assess its ability to remove bias due to genomic pre-selection of young sires. In the study of Patry and Ducrocq [10], actual data were used to simulate breeding values and mimic genomic pre-selection of the last generation of sires to assess bias in national BLUP evaluations. In the current study, the same population and simulated data as in [10] were used to measure bias before and after including genomic information. In addition, the issue of combining genomic with traditional information, i.e. phenotypes and pedigree, is addressed.

## Methods

### Overview

Data were generated as described in Patry and Ducrocq [10] and GEBV were simulated for a cohort of young sires that was considered as a cohort of selection candidates. GEBV were used to retain a proportion of the best candidates, mimicking genomic pre-selection. To account for this selection step in BLUP evaluations at the national level, GEBV were de-regressed to provide genomic pseudo-performances for all the genotyped candidates. A weight derived from the increase in reliability of EBV due to genotype information was associated to each pseudo-performance. Pseudo-performances and their associated weights were included in Henderson's mixed model equations as if they were regular records. Three scenarios were compared to a situation without pre-selection. Each scenario corresponded to a different type and/or amount of information included in the evaluation: actual performances of selected young sires only or combined with de-regressed genomic pseudo-performances, for the selected or all the candidate sires. Bias and accuracy of BLUP evaluations were measured for each scenario.

### Populations and cohorts of the study

In their study [10], Patry and Ducrocq used actual pedigree records and records from the 2008 national type trait evaluations for the Holstein breed in France to simulate breeding values of selection candidates. The animals of interest were defined as the youngest progeny-tested bulls with no second crop daughters, hereafter called young sires (YS). Their daughters and the dams of their daughters were also known. Two populations were considered for BLUP evaluations, one in which progeny testing was carried out (CONTROL population) and one reflecting genomic pre-selection in the last generation (GPS population). To mimic genomic pre-selection among YS, GEBV were generated together with true breeding values (TBV) in the GPS population. GEBV of full-sib families of candidate sires were generated. Among each full-sib family, it was assumed that the sib with the highest GEBV was selected, while the remaining full-sibs were culled. In the CONTROL population, only TBV were simulated for YS. As with the real datasets, only selected sires had daughters, and their performances were simulated. In the current study, as in [10], the same cohorts and sets of data were used, including GEBV and TBV for all candidate sires, and performances for their daughters.

### Data generation: TBV, GEBV, performances

For young sires, TBV and GEBV were simulated jointly (in the GPS population) from multivariate normal distributions and conditional on parent average (EBV before including progeny information). Variances and covariances of the distributions depended on the genetic variance of the trait and reliabilities of genetic and genomic evaluations. Direct genomic reliability and pedigree reliability were distinguished. Reliability of GEBV was defined as a combination of genomic and pedigree-based information. Pedigree reliabilities were obtained from the true data analysis before including progeny information. Direct genomic reliabilities were computed assuming the genomic contribution contributed *n *additional daughter records. Various values of *n *were used in the simulations. Daughter performances were computed using estimated fixed effects from the true data analysis, simulated TBV of YS, and the distribution of dam EBV. For more details, see Patry and Ducrocq [10]. Simulations were replicated 50 times.

### Estimation of breeding values

Breeding values were estimated for all the animals in both populations, CONTROL and GPS, based on daughter performances and pedigree-based information and using BLUP applied to a single-trait animal model. In the CONTROL population, EBV of YS were unbiased [10]. In the GPS population, only pre-selected YS had daughters and therefore, only their performances were available for the BLUP evaluation. Genomic pre-selection was not taken into account in the estimation of breeding values by BLUP and EBV of YS were shown to be biased [10].

### Computation of de-regressed GEBV

To account for the genomic selection step in BLUP evaluations at the national level, GEBV were de-regressed as described in the following paragraph and weighed by the increase in reliability due to genomic information. Estimated breeding values $\hat{a}$ are usually obtained as solutions of the MME:

(1) $$\left[\begin{array}{cc} X^{\prime }R^{-1}X & X^{\prime }R^{-1}Z\\ Z^{\prime }R^{-1}X & \;Z^{\prime }R^{-1}Z+A^{-1}\alpha \end{array}\right]\left[\begin{array}{c} \hat{\beta }\\ \hat{a} \end{array}\right]=\left[\begin{array}{c} X^{\prime }R^{-1}y\\ Z^{\prime }R^{-1}y \end{array}\right]$$

where **β **and **a **are vectors of fixed effects and breeding values, **A **is the additive genetic relationship matrix, **X **and **Z **are incidence matrices assigning observations to effects, and α is the variance ratio between residual and genetic variance $\left(\alpha =\sigma_{e}^{2}∕\sigma_{a}^{2}\right)$. From (1), EBV $\overset{}{â}$ can be computed from:

(2) $$\left(Z^{\prime }R^{-1}Z+A^{-1}\alpha )\hat{a}=Z^{\prime }R^{-1}(y-X\hat{\beta }\right)$$

This equation is obtained after correction for the breeding value of their dam and absorption of each daughter equation, such that only equations corresponding to sires and their ancestors are left.

In a regular de-regression procedure, as described by Jairath et al. [11], the **EDP **vector is obtained from the right hand side of:

(3) $$(EDC+A_{s}^{-1}\alpha )\hat{a_{s}}=EDC.EDP$$

where **EDC **is a diagonal matrix of Effective Daughter Contributions with element *EDC_i _*representing the amount of information coming from daughter phenotypes for each sire *i*. **EDP **is a vector of de-regressed proofs also called Effective Daughter Performances; and **A**_s _and **a**_s _are the numerator relationship matrix and the vector of breeding values of the sires and their ancestors. Assuming that **a**_s _is known from the solution of (1) or (2), we have:

(4) $$EDP={\left(EDC\right)}^{-}(EDC+A_{s}^{-1}\alpha )\hat{a_{s}}$$

Equation (4) can be adapted to compute for each genotyped sire *i*, a "genomic" pseudo-performance $EDP_{i}^{g}$, similar to the effective daughter performance *EDP_i _*.

Let ΔRel*_i _*be the increase in reliability of DGV(Direct Genomic Value) or GEBV for sire *i *compared to its classical EBV. It will be referred to as the "direct genomic reliability": $\Delta \text{Re}\;{\text{l}}_{i}=\frac{{\text{EDC}}_{i}^{g}}{{\text{EDC}}_{i}^{g}+\text{k}}$ or equivalently: ${\text{EDC}}_{i}^{g}=\frac{\text{k}\Delta \text{Re}1_{i}}{1-\Delta \text{Re}1_{i}}$ where ${\text{EDC}}_{i}^{g}$ is the "genomic" effective daughter contribution, $\text{k}=\frac{4-{\text{h}}^{2}}{{\text{h}}^{2}}$ and h^2 ^is the heritability of the trait. Replacing in **a**_s _equation (4) by **g **, the vector of GEBV, it follows that the vector **EDP**^g ^of genomic pseudo-performances is the solution of:

(5) $$(EDC^{g}+A_{s}^{-1}\alpha )\;\hat{g}=EDC^{g}.EDP^{g}$$

Note that vector **g **does not only include GEBV for genotyped animals but also GEBV for non-genotyped ancestors. **g **was split into two vectors (**g***_g _*, **g***_ng _*) distinguishing genotyped animals (*g*) from non genotyped ones (*ng*). After appropriate reordering of rows and columns, let:

(6) $$A_{s}^{-1}=\left[\begin{array}{cc} A^{gg} & A^{gng}\\ A^{ngg} & A^{ngng} \end{array}\right]$$

Assuming $EDP_{i}^{g}$ equal to zero for non genotyped sires, vector **g***_ng _*is computed solving the following equation:

(7) $$A^{ngng}{\hat{g}}_{ng}=-A^{ngg}{\hat{g}}_{g}$$

This de-regression procedure removes the parent average effect. Therefore, either GEBV which include a residual polygenic effect or DGV can be used in **g **.

To be able to include the genomic pseudo-performances in a national genetic evaluation, sire **EDP**^g ^and **EDC**^g ^must be adapted to an animal model, where the sire variance used in a sire model is replaced by the additive genetic variance. This is done by multiplying **EDP**^g ^by 2 and by multiplying **EDC**^g ^by $\frac{\alpha }{\text{k }}$ [12].

### Inclusion of genomic pseudo-performances into BLUP evaluations

For the GPS population, three different datasets of performances were created to obtain BLUP evaluations, leading to three scenarios to account for genomic pre-selection at the national level:

1. BLUP evaluations included only one type of phenotypes for the YS, i.e. the simulated performances of their daughters. These "actual" phenotypes were available only for the YS which were pre-selected based on their genomic information. Thus culled YS were not included in the evaluation. We called this scenario "GPS_no" and has been shown to result in biased EBV [10].

2. BLUP evaluations included two types of phenotypes for the YS, i.e. the simulated performances used in scenario GPS_no and the genomic pseudo-performances **EDP**^g^, i.e. the de-regressed GEBV derived above, but **EDP**^g ^were only available for the selected candidates. This scenario was called "GPS_sel".

3. BLUP evaluations used the same two types of phenotypes available for the pre-selected YS as in the previous scenario but this time, the genomic pseudo-performances **EDP**^g ^were also included for candidates culled after the genomic pre-selection step. This scenario was called "GPS_all". Hence all candidate sires have an associated pseudo-performance.

### Sets of parameters

Different levels of trait heritability, different proportions of retained young sires after genomic selection and different accuracies of GEBV were used to define several parameter sets, as in Patry and Ducrocq [10]. Thus, two type traits, udder depth (UD) and foot angle (FA), were considered because of their contrasted heritabilities (0.36 versus 0.14). The genetic variance was 0.25 for UD and 0.14 for FA. Seven hundred and ninety-nine selected YS and a total of 40,222 daughters with UD records and 601 selected YS and their 31,976 daughters with FA records were identified. Two proportions of selected YS were tested: 10% and 25%. For example, when 10% of YS were retained after genomic selection, 7,990 pairs of TBV and GEBV for UD were simulated to identify, after proper ranking, 799 selected YS and 7,191 culled YS. We assumed an initial value of 10 effective daughter records so that the direct genomic reliability was 0.50 for UD and 0.26 for FA. Because of the lower heritability of FA, we also tested a value of 26 EDC to achieve a direct genomic reliability of 0.50, as for UD. See Table 1 for the definition of all the parameter sets. Depending on the set of parameters and on the scenario (GDP_no, GDP_sel and GDP_all), a different number of actual daughter performances and genomic pseudo-performances were included, see Table 2.

**Table 1:** Size of the cohorts according to different levels of heritability and genomic selection intensity

Proportion of selected young sires	10%			25%		
**Traits**	**Full-sibs family size**	**Number of selected young sires (and their daughters)**	**Number of culled young sires (without daughters)**	**Full- sibs** **Family size**	**Number of selected young sires (and their daughters)**	**Number of culled young sires (without daughters)**

Udder depth (h^2 ^= 0.36)	7990	799 (40222)	7191	3196	799 (40222)	2397

Foot Angle (h² = 0.14)	6010	601 (31976)	5409	2404	601 (31976)	1803

**Table 2:** Number and type of performances available in BLUP evaluations for the four tested scenarios

Proportion of sires retained after genomic selection		25%		10%	
**Performances**		**Actual daughter records**	**Genomic pseudo-performances**	**Actual daughter records**	**Genomic pseudo- performances**

UD^d ^trait	After progeny testing	40222	0	40222	0
	
	After genomic pre-selection: GPS_no^a^	40222	0	40222	0
	
	GPS_sel^b^	40222	799	40222	799
	
	GPS_all^c^	40222	3196	40222	7990

FA^e ^trait	After progeny testing	31976	0	31976	0
	
	After genomic pre-selection: GPS_no^a^	31976	0	31976	0
	
	GPS_sel^b^	31976	601	31976	601
	
	GPS_all^c^	31976	2404	31976	6010

^a^genomic pre-selection of young sires but no inclusion of genomic pseudo-performances; ^b^genomic pre-selection of young sires and genomic pseudo-performances were included for selected young sires; ^c^genomic pre-selection of young sires and genomic pseudo-performances were included for all candidate sires; ^d^udder depth; ^e^foot angle

### Statistical analysis of the data

National BLUP evaluations were performed in the four situations presented in Table 2. Breeding values were estimated in the CONTROL population and under the three scenarios in the GPS population (GPS_no, GPS_sel, GPS_all). Before further statistical analysis of the resulting EBV, all EBV were expressed in genetic standard deviation units of the trait (σ_G_). The mean Mendelian sampling term was estimated as the mean difference between the young sires' EBV and their parent average across all the YS included in each scenario. This estimate indicated how much the usual MME assumption of zero expectation for the Mendelian sampling term was violated. As in Patry and Ducrocq [10], three indicators were used to assess the quality of BLUP evaluations and were compared among the four scenarios: bias, true reliability (ρ²) and mean square error (MSE), as defined below. Let *a_i _*and *a_i _*be respectively the TBV and EBV of each young sire *i *in each replicate *r*.

(8) $$bias=\frac{1}{50}\sum_{r=1}^{50}\left(\frac{1}{n}\sum_{i=1}^{n}\left(\hat{a_{i}}-a_{i}\right)\right)$$

(9) $$\rho^{2}={\left(\frac{1}{50}\sum_{r=1}^{50}\frac{cov(\hat{a_{r}},a_{r})}{\sqrt{var(\hat{a_{r}})var(\hat{a_{r}})}}\right)}^{2}$$

(10) $$MSE\;=\frac{1}{50}\sum_{r=1}^{50}(Var(\hat{a_{r}}-a_{r})+{\left(\hat{a_{r}}-a_{r}\right)}^{2})$$

True reliability and MSE characterize the accuracy of BLUP evaluations. Statistics were computed for two groups of interest, the young sires and their daughters and averaged over the 50 replicates. For both groups and each scenario, they were calculated for all animals actually included in the BLUP evaluations: both eliminated and selected candidates were analysed in the CONTROL and GPS_all scenarios whereas only selected candidates were included in the analysis of GPS_no and GPS_sel scenarios.

## Results

### Including information on all the selection candidates avoids pre-selection bias

To illustrate the bias process and the approach to account for pre-selection, only the results for the evaluation of UD (h² = 0.36) when 25% of the YS were retained based on their GEBV will be presented. In the CONTROL population, the EBV of YS were unbiased since all the selection candidates were included in the BLUP evaluation (Table 3): both the mean Mendelian sampling estimate and the mean difference between true and estimated breeding values were not significantly different from zero. In contrast, the mean Mendelian sampling estimate and the bias were significantly different from zero, true reliability decreased and MSE increased, when genomic pre-selection of sires was applied (GPS population) but not accounted for in the evaluation (GPS_no scenario). When genomic pseudo-performances were included for selected sires only (GPS_sel scenario), the true reliability of BLUP evaluations increased compared to the scenario GPS_no due to the explicit addition of genomic information to the traditional pedigree and performance information. The MSE also decreased, indicating that the quality of BLUP evaluations was improved. However, the bias was still significantly different from zero (Table 3). The genomic selection process was completely accounted for only when genomic pseudo-performances for culled sires were also included in the evaluation model (GPS_all scenario). In this case, the mean Mendelian sampling estimate and the bias of the cohort of selected sires were not significantly different from zero. Including de-regressed GEBV for all YS in the evaluation model as in GPS_all scenario not only accounted for genomic pre-selection, contrary to the GPS_sel scenario, but also increased accuracy of BLUP evaluations compared to the GPS_no scenario.

**Table 3:** Quality of BLUP evaluations of young sires for udder depth after a 25% genomic pre-selection

Scenarios	Mendelian sampling estimate (in *σ*_g_^d^)	Bias (in *σ*_g_^d^)	True reliability	Mean square error
CONTROL	-0.001 (ns)	0.002 (ns)	0.756	0.183

GPS_no^a^	0.304 (***)	-0.146 (***)	0.727	0.188

GPS_sel^b^	0.188 (***)	-0.138 (***)	0.763	0.165

GPS_all^c^	-0.003 (ns)	-0.019 (ns)	0.760	0.150

H0 = {μ = 0}: ns = non significant (p > 0.001); *** = p-value < 0.001; ^a^genomic pre-selection of young sires but no inclusion of genomic pseudo-performances; ^b^genomic pre-selection of young sires and genomic pseudo-performances were included for selected young sires; ^c^genomic pre-selection of young sires and genomic pseudo-performances were included for all candidate sires; ^d^genetic standard deviation of the trait

### Influence of heritability and pre-selection intensity

Previous research showed that, when the trait heritability is lower or the genomic pre-selection intensity is higher, the relative magnitude of the bias due to genomic selection increases when the genomic pre-selection intensity is not accounted for in the evaluation model [10]. The average bias and MSE are presented in Table 4 for YS and in Table 5 for their daughters for different combinations of trait heritability and genomic pre-selection intensity levels and when selection based on genomic information is fully (GPS_all) or not accounted for (GPS_no). For the YS cohort (Table 4) in the GPS_no scenario, the bias ranged from -0.146 to -0.338 σ_G_, and from -0.03 to 0 σ_G _in the GPS_all scenario. In the latter case, the bias was also almost zero in the cohort of daughters (Table 5). Regardless of the magnitude of the initial bias for YS or their daughters, including genomic pseudo-performances for all the selection candidates provided the MME with sufficient information on the selection process to effectively reduce the bias.

**Table 4:** Quality of BLUP evaluations with or without accounting for pre-selection in the cohort of selected young sires

Heritability	Proportion of selected young sires	Bias (in *σ*_g_^c^)		Mean squared error	
		
		GPS_no^a^	GPS_all^b^	GPS_no^a^	GPS_all^b^
0.36 (UD^d ^trait)	10%	-0.227 (***)	-0.030 (*)	0.217	0.157
	
	25%	-0.146 (***)	-0.019 (ns)	0.188	0.150

0.14 (FA^e ^trait)	10%	-0.338 (***)	-0.020 (ns)	0.364	0.222
	
	25%	-0.214 (***)	-0.011 (ns)	0.305	0.229

H0 = {μ = 0}: ns = non significant (p > 0.001); *** = p-value < 0.001; ^a^genomic pre-selection of young sires but no inclusion of genomic pseudo-performances; ^b^genomic pre-selection of young sires and genomic pseudo-performances were included for all candidate sires; ^c^genetic standard deviation of the trait; ^d^udder depth; ^e^foot angle

**Table 5:** Quality of BLUP evaluations with or without accounting for pre-selection in the cohort of daughters of the selected young sires

Heritability	Proportion of selected young sires	Bias (in *σ*_g_^c^)		Mean squared error	
		
		GPS_no^a^	GPS_all^b^	GPS_no^a^	GPS_all^bb^
0.36 (UD^d ^trait)	10%	-0.074 (***) -0.009 (ns)		0.547	0.541
	
	25%	-0.044 (**) -0.002 (ns)		0.544	0.540

0.14 (FA^e ^trait)	10%	-0.144 (***) -0.010 (ns)		0.685	0.662
	
	25%	-0.092 (***) -0.006 (ns)		0.674	0.660

H0 = {μ = 0}: ns = non significant (p > 0.001); *** = p-value < 0.001; ^a^genomic pre-selection of young sires but no inclusion of genomic pseudo-performances; ^b^genomic pre-selection of young sires and genomic pseudo-performances were included for all candidate sires; ^c^genetic standard deviation of the trait; ^d^udder depth; ^e^foot angle

### Impact of genomic evaluation accuracy

In the previous situations, we considered diagonal values of **EDC**^g ^of 10 for UD and 26 for FA. In Table 6, we compared these results for FA with a situation where diagonal values of **EDC**^g ^were assumed to be 10 instead of 26; hence the accuracy of the genomic evaluations was assumed to be lower. In this case, the expected genetic gain genetic trend was smaller and selection was less efficient. As a result, the bias due to not accounting for pre-selection (GPS_no) was smaller than with an **EDC**^g ^of 26. However, the bias was also less reduced by including genomic pseudo-performances for selected YS (GPS_sel) when EDCg was equal to 10. This illustrates the fact that the accuracy of GEBV is a key element when including genomic performances for all candidates in the evaluation model to account for bias due to genomic pre-selection.

**Table 6:** Effect of accuracy of genomic evaluations on BLUP evaluations for foot angle in the cohort of selected young sires

EDC^g d^	Proportion of selected young sires	Bias (in *σ*_g_^c^)		Mean squared error	
		
		GPS_no^a^	GPS_all^b^	GPS_no^a^	GPS_all^b^
10	10%	-0.249 (***)	-0.098 (***)	0.339	0.270
	
	25%	-0.155 (***)	-0.054 (**)	0.299	0.257

26	10%	-0.338 (***)	-0.020 (ns)	0.305	0.222
	
	25%	-0.214 (***)	-0.011 (ns)	0.364	0.229

H0 = {μ = 0}: ns = non significant (p > 0.001); *** = p-value < 0.001; ^a^genomic pre-selection of young sires but no inclusion of genomic pseudo-performances; ^b^genomic pre-selection of young sires and genomic pseudo-performances were included for all candidate sires; ^c^genetic standard deviation of the trait; ^d^effective daughter contribution from genomic EBV

## Discussion

The inclusion of a genomic pseudo-performance, i.e. a de-regressed GEBV, for all genotyped candidates reduced the GEBV bias to (almost) zero in most simulated situations, regardless of the genomic selection intensity. Inclusion of genomic pseudo-performance resulted in a better description of the genetic characteristics of the population of candidates. Consequently, the overall average Mendelian sampling term had a zero expectation and the classical assumptions of the BLUP model were more closely met. However, the results showed that the effectiveness of this approach depended on the quality of genomic evaluations. This approach was more effective for traits with a higher heritability or for genomic evaluations with a higher accuracy. As expected, adding genomic data increased the amount of information contributed to the genetic evaluation and this information was distributed to relatives through the additive relationship matrix. In fact, including genomic pseudo-performance is not as straightforward as adding regular performance to BLUP evaluations [13]: obviously, accuracy of EBV increases as the number of daughters increases but this is not always the case with an increasing number of genotyped animals. Indeed, genotyped parents correctly add information to non-genotyped progeny and genotyped progeny contribute information to non-genotyped parents but the total amount of additional information from genotyped relatives cannot exceed the gain in accuracy from genotyping the animals themselves [8]. Furthermore, if a progeny and its sire are both genotyped, the progeny genotype does not provide any additional information to the sire and vice versa [6]. Thus including without care genomic pseudo-performances for both the sire and its progeny will result in double counting genomic contributions, once directly, and once via relatives through the additive relationship matrix [8]. Therefore, BLUP evaluations must account for such data redundancy.

In this study, only YS were genotyped and we implicitly assumed that none of their sires were from the reference population, hence avoiding the issue of redundant genomic information and overestimated reliability of genomic evaluation [14]. However, in a more realistic case, the weight of genomic information might be overestimated by **EDC**^g ^and a tailored reduction of **EDC**^g ^should be implemented. Nevertheless, despite the simplified assumptions and computations, the approach used was shown to be promising and demonstrated that including information on culled candidates is essential.

With the addition of genomic information, inflated reliabilities have been reported regardless of the method used to blend genomic and traditional information: the selection index approach [2,14], the single-step approach [4], or the current approach, which was initially proposed by Ducrocq and Liu [6]. Some strategies have been suggested to prevent the reliability of genotyped animals from approaching 1. Ducrocq and Liu [6] have proposed an iterative approach adapted from the information source method [15] to compute reliability from genomic information. In their situation, the **EDC**^g ^were derived under constraints such that the final genomic contribution to reliabilities was bounded. The reliabilities of GEBV appeared to be reasonable. However, the issue was not completely solved since reliabilities were still overestimated for sires with many genotyped progeny [6]. Mäntysaari and Strànden have proposed to use a multi-trait evaluation to combine DYD and DGV, where DGV are treated as an indicator trait with a high correlation to the considered trait. Then, reliabilities of GEBV are naturally bounded to the square of this correlation so that genomic relationships are less overestimated. Such a correlation between EBV and DGV or GEBV could be estimated following the method proposed by Kachman [17] and implemented by MacNeil et al.[18].

The single-step approach [4,5] offers an appealing solution in the sense that genomic, phenotypic and pedigree information are analyzed simultaneously. However, unless it is assumed that all the genetic variation is described by the SNP markers, these procedures face the problem of finding an appropriate weighting of genomic and pedigree-based information [4,5]. In some studies, the lack of independency between the three sources of information (genomic, phenotypic, pedigree based) has been considered through a scaling of the residual variance [16,19] but only approximate solutions have been developed so far. Further appropriate developments are necessary to better compute **EDC**^g ^and to improve the method of including genomic performances in BLUP evaluation to account for bias due to genomic pre-selection. The approach presented here involves an additional step, before running national BLUP evaluations, i.e. computation of genomic pseudo-performances. This step is easy to implement as de-regression is commonly used, like in routine international genetic evaluations [11]. This method has several key advantages. First, it is independent from the methodology used to predict genomic EBV (GBLUP, Bayesian methods, etc), secondly, it can be applied to different evaluation models without further developments and, finally, the size of the genotyped population is not a constraint.

With the current breeding schemes in dairy cattle, a period of about four years is necessary between the genomic pre-selection step and the introduction of the first records of daughters in BLUP evaluations. Since genomic selection has begun more than two years ago in several countries, the first biased evaluations may occur within the two next years. Thus the need to implement an easy to apply approach to account for genomic pre-selection is urgent. The approach proposed here requires only limited modifications (if any) of the existing national evaluation software. However, further work is needed to control the dependency between BLUP evaluations and genomic evaluations. To account for genomic pre-selection, EBV must include genomic information and these unbiased EBV are then used as input for future equations for genomic predictions. The issue is that genomic information will be double counted when computing GEBV. One way to circumvent this problem would be to iterate between the classical genetic and genomic evaluations.

Two alternatives, both potentially problematic, are possible: on the one hand, genomic pre-selection of young sires leads to biased EBV and therefore to biased DYD which are then used to update genomic predictions. On the other hand, incorporating genomic records into national BLUP evaluations inflates the accuracy of BLUP EBV of some animals and makes classical genetic and genomic evaluations dependent from each other. Thus, a compromise has to be found between the use of biased EBV on one side, and double counting of genomic information and overestimation of reliabilities on the other side.

In this study, the underlying context was rather optimistic. In particular, it was assumed that all data from selected and culled candidates were available at the national level. For example, the use of pre-selected bulls from foreign breeding schemes was not considered. Moreover, in the context of national and international competition, breeding companies may be reluctant to release information on their selection strategy and objectives, and may not be willing to share data on culled animals. Our study clearly shows that this would be very detrimental for at least three reasons: first, EBV of pre-selected bulls would be underestimated; secondly, the resulting bias would be transferred to the rest of the population (e.g., daughters) in an uncontrolled way; and finally, genomic predictions using results from these biased evaluations would be sub-optimal. Therefore, it is essential that information originating from current implementations of genomic selection (GEBV of all animals, or at least selection differentials) at least be shared at the national level. Ignoring genomic pre-selection at the national level impacts national EBV and, as a consequence, international EBV too. We are currently investigating to what extent the transmission of biased or unbiased national EBV for selected bulls only could bias international genetic evaluations.

## Conclusions

There is an urgent need to account for genomic pre-selection of young sires before their national EBV become biased. Based on a real dairy cattle dataset, breeding values were generated in the last generation of sires to mimic genomic pre-selection. In this study, including a genomic pseudo-performance based on GEBV for all the selection candidates strongly reduced or removed biases, regardless of their magnitude. However, this approach does not account for some potential overestimation of the weight that is placed on genomic information and for dependency of genetic and genomic evaluations. Thus, the proposed method may need further improvement, but in the short term, it makes possible to implement a simple and general procedure that accounts for these new selection practices in BLUP evaluations at the national level. In addition, this approach provides an alternative method to combine genomic, phenotypic and pedigree data in multiple steps procedures which is easy to understand and implement.

## Competing interests

The authors declare that they have no competing interests.

## Authors' contributions

CP implemented the proposed methodology and participated in the results analysis.

VD conceived the study, participated to its implementation and in the analysis. CP and VD were both involved in drafting the manuscript. They both read and approved the final manuscript.
